# The thermodynamic and life-cycle assessments of a novel charging station for electric vehicles in dynamic and steady-state conditions

**DOI:** 10.1038/s41598-023-38387-0

**Published:** 2023-07-10

**Authors:** Hossein Pourrahmani, Chengzhang Xu, Jan Van herle

**Affiliations:** Group of Energy Materials, École Polytechnique Fédérale de Lausanne, 1951 Sion, Switzerland

**Keywords:** Environmental impact, Batteries, Fuel cells, Renewable energy

## Abstract

The current study performs the thermodynamic and life-cycle assessments (LCA) of a novel charging station in two system designs. The goal is to design an efficient charging station for electric vehicles with high efficiencies and low environmental impacts using Solid Oxide Fuel Cell (SOFC) technology. SOFC is considered a sustainable and environmentally friendly technology to generate electricity compared to combustion engines. To ameliorate the performance, the exhaust heat of the SOFC stacks will be recovered for hydrogen production in an electrolyzer. The system uses four SOFCs to charge the electric vehicles while the output heat is recovered by an Organic Rankine Cycle (ORC) to generate further electricity for hydrogen production in an electrolyzer. In the first design, it is assumed that the SOFC stacks will work full-load during the 24 h of the day, while the second design considers full-load operation for 16 h and part-load (30%) operation for 8 h. The second design of the system analyzes the possibility of integrating a ${\mathrm{LiMn}}_{2}{\mathrm{O}}_{4}$ lithium-ion battery stores the excessed electricity once the power load is low and acts as a backup in high power demands. Results of the thermodynamic analysis calculated the overall efficiencies of 60.84% and 60.67% for the energy and exergy, respectively, with the corresponding power and hydrogen production of 284.27 kWh and 0.17 g/s. It was observed that higher current density would increase the output of SOFC while reducing the overall energy and exergy efficiencies. In dynamic operation, the use of the batteries can well balance the change of the power loads and improve the dynamic response of the system to the simultaneous changes in the power demand. LCA results also showed that the 284.27kWh system leads to global warming (kg ${\mathrm{CO}}_{2}$ eq) of 5.17E+05, 4.47E+05, and 5.17E+05 using Solid Oxide Electrolyzer (SOE), Proton Exchange Membrane Electrolyzer (PEME), and Alkaline Electrolyzer (ALE), respectively. In this regard, the usage of PEME has the lowest impact on the environment in comparison to SOEC, and ALE. A comparison between the environmental impacts of different ORC’s working fluids also suggested against the usage of R227ea while R152a showed promising results to be used in the system. The size and weight study also revealed that the battery benefits from the lowest volume and weight in comparison to the other components. Among the considered components in this study, the SOFC unit and the PEME have by far the highest volume.

## Introduction

With the current advances in electric vehicles (EVs), the needed infrastructure in addition to the policies should be improved to accelerate the large scale deployments^1^. One of the main barriers to further commercializing EVs is the lack of charging stations throughout the world^2^. Selecting the right technology to generate the required electricity is still debatable^3^. For example, as a case study, the demand for EVs in Scandinavia-Germany has been provided by wind power and thermal power using a cost-minimization investment model^4^. The candidate technology should be efficient and environmentally friendly to be a promising option for further investments. Additionally, a concentrated effort should be made on the operational conditions of the delivery system to optimize the performance as mentioned by Jayachandran et al.^5^. The use of fuel cells in a charging station can be an interesting choice since the transportation loss of gas is much smaller than that of electricity.

Fuel cells are electrochemical devices, which produce electricity in an environmentally friendly manner^6^. Fuel cells are considered competitive alternatives for fossil-based devices due to lower emissions and better efficiencies and they have overall advantage over batteries in the terms of energy density as mentioned by Ref.^7^. Malik et al.^8^ performed a comparative study to emphasize that Solid Oxide Fuel Cells (SOFCs) with an operating temperature range of 800 °C to 1200 °C are mainly being used for stationary applications while Proton Exchange Membrane Fuel Cells (PEMFCs) are more appropriate for mobility purposes. High temperature operation of the SOFCs enables them to have a more flexible choice of fuels such as ammonia and biogas as mentioned by Fuerte et al.^9^ and Saadabadi et al.^10^, respectively. As heat is also being produced during the working process of a SOFC, integrating a cycle to re-use the SOFC’s exhaust heat is of interest to designing more efficient integrated systems.

Different bottoming cycles can be used in combined systems to improve the efficiency and the performance^11^. Zhang et al.^12^ indicated that among the introduced bottoming cycles, the Organic Ranking Cycle (ORC) is proved to be more efficient in comparison to the other available alternatives. Hereafter, ORC benefits from the possibility to recover heat at a comparatively low temperature range of 80 °C to 350 °C^13^. Thus, the integration of this cycle to recover the waste heat of the SOFC units can drastically improve the overall efficiencies. Aliahmadi et al.^14^ used an ORC cycle to recover the heat from geothermal sources and achieve an exergy efficiency of around 60%. Asghari et al.^15^ used ORC to recover the heat of SOFC, which is used to supply the power for the absorption chiller for cooling purposes. In similar studies by Pourrahmani et al., the waste heat of the SOFC units were recovered using ORC^16^ and the Absorption refrigeration system^17^ to provide power and cooling, respectively. Although the addition of the bottoming cycles will ameliorate the energy efficiency, the exergy efficiency, environmental effects, costs, size, weight, and other parameters should be analyzed in detail to be considered for charging station applications. Also, the ORC’s generated electricity can be directly used in the charging station or utilized to produce hydrogen in an electrolyzer. Although a SOFC-based charging station has been proposed in the literature^16^, further waste heat recovery of the SOFC unit can be done using the ORC and electrolyzer units, which have not been analyzed before.

Among different types of electrolyzers, the Proton exchange membrane electrolyzer (PEME) is considered the most commercialized type with the notable advantages such as large current density, high hydrogen purity, and great conversion efficiency^18^. The PEME unit can be combined with other cycles to produce hydrogen in co-generation systems. Designing a charging station that provides electricity for EVs enables a better transition from fossil-fuel-based vehicles to environmentally friendly alternatives. As mentioned by Al Wahedi et al.^19^, the efficient design of the charging station also demands the integration of a storage unit to save the excessed electricity in low power demands and provide power in high electricity demands.

The application of batteries in power generation systems is mostly related to the storage/backup system in addition to accelerating the charing process, which was analyzed by Deng et al.^20^. The usage of batteries enables the more efficient performance of the other power-generating components in the system. For example, in the case of solar photovoltaic panels, batteries can store the received energy from the sun and enable the performance of the system by providing electricity at night. In the case of charging stations, the power demand may be lower in some periods during the day, hence batteries can be used as the backup/storage systemin electric power systems^21^. Characterization of the dynamic and LCA performances of the system enables the authorities for better decisions. Although using fuel cell technology and ORC may be considered a more interesting option in comparison to fossil fuel-based technologies to generate electricity, a detailed life cycle assessment (LCA) is also needed. Although in a previous study by the authors^17^, a SOFC-based charging station, that also provided cooling using an absorption system, was proposed and evaluated from different aspects, the role of the electrolyzer unit, different types of SOFC fuels, and ORC’s working fluids were not investigated on the environmental impacts of the system and the performance of the system. Additionally, the current study benefits from a detailed LCA, weight, and size analysis that differentiate the current study with the existing knowledge in the literature.

Using LCA, the environmental impacts of a commercial product in all the possible stages of its life cycle can be determined^22^. For example, in the case of SOFC, the injected fuel into the system can be different, which changes the environmental impacts of SOFC consequently. Performing the LCA for the proposed system can determine the environmental impacts on human health, the ecosystem, and resources^23^. Furthermore, it can provide useful information on the effects of the system on global warming, water consumption, ionizing radiation, Ozone formation, etc.^24^. In this regard, performing LCA analysis for the current suggested system can fill the existing gap in the research studies to provide adequate information for decision-makers.

### Novelties of the current research

This study aims to suggest the efficient design of a charging station for electric vehicles. Thus, a cogeneration system with four SOFC stacks and an ORC unit as the waste heat recovery has been suggested with and without a storage unit. Although this charging station aims to provide electricity for EVs, the produced hydrogen in the considered electrolyzer can supply the required hydrogen for fuel cell electric vehicles as well. The PEME will use the generated electricity by the ORC, which recovers the exhaust heat of the SOFC stacks. In the first step, the system is analyzed without the backup/storage unit. Then, a dynamic characterization will be performed including the batteries. Once the batteries are combined, three SOFC stacks will go through partial operation at night as the ${\mathrm{LiMn}}_{2}{\mathrm{O}}_{4}$ Lithium-ion battery can provide the required electricity. Furthermore, the system is analyzed by energy and exergy characterizations to calculate the overall efficiencies, output power, and hydrogen production. Furthermore, the environmental impacts of the suggested integrated systems have been analyzed with LCA considering different types of fuels for the SOFC stacks, working fluids for the ORC, and technologies for the electrolyzer unit. In summary, the novelties of the current study can be categorized as follows:

- Proposing a novel design for the charging stations using SOFC technology as the prime movers followed by heat recovery by the ORC and electrolyzer units. Three different types of electrolyzers were analyzed once integrated into the other components and the environmental impacts are analyzed. The required equations to perform the thermodynamic analysis were derived considering the integrated electrolyzer.
- Making concentrated efforts on the environmental impacts of the proposed system considering the different types of fuels for the SOFC unit. Additionally, the environmental impacts of different types of electrolyzers have been analyzed and discussed. The important role of selecting the right ORC’s working fluid has been mentioned through a detailed LCA analysis.
- Evaluating the size and the weight of the suggested system to reach a reasonable performance with a compact system.
- The dynamic response of the system considering the changes in the power demand of the charging station and the respective impacts on the performance of the electrolyzer and the heat recovery system.

## Problem description

**Figure 1:**
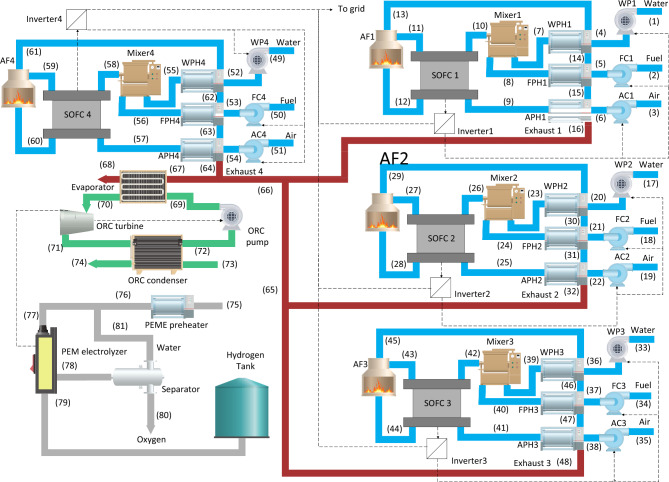
The details of the steady-state cogeneration system to produce hydrogen and electricity as the charging station for electric vehicles. (The figure has been obtained using OriginPro, Version *9.9*, 2022. OriginLab Corporation, Northampton, MA, USA, available at: https://www.originlab.com/).

In the current suggested multi-generation system, the main aim is to produce electricity for EVs using SOFC stacks. Thus, four SOFC stacks are being integrated to generate the required electricity for EVs. The wasted heat of these four stacks is transferred to an ORC to be converted to electricity. Figure 1 illustrates the detailed schematic of the charging station indicating the thermodynamic states in the steady-state condition. Each SOFC stack provides the required electricity for its compressors, and pump, then the excess power will be used to charge the EVs. ORC turbine also generates the needed electricity for the ORC pump in addition to the required electricity for the PEME. Here, a PEME has been used to provide hydrogen by the produced electricity of the ORC unit. In other words, the wasted heat of the SOFC stacks is re-used by the ORC unit, and electricity is generated that is an input for the PEME.

Regarding Fig. 1, it should be considered that four different SOFC stacks operate independently. The operation of each SOFC stack demands the usage of pre-heaters, pumps, compressors, etc., hence four different SOFC units operate independently. Each SOFC unit includes a SOFC stack, three pre-heaters, two compressors, a pump, a mixer, an inverter, and an afterburner. As stated in Fig. 1, the four SOFC units in addition to the ORC unit, and the PEME unit, create the designed integrated system to act as the charging station for the EVs. This study will model all the indicated components of the integrated system in Fig. 1, including the SOFC unit (including the SOFC stack, compressors, pre-heaters, pump, mixer, afterburner, and inverter), the PEME unit (including the PEM electrolyzer, PEM pre-heater, separator, and hydrogen tank), and ORC unit (including the evaporator, turbine, pump, and condenser) and evaluate the performance considering the energetic, exergetic and environmental aspects.

**Figure 2:**
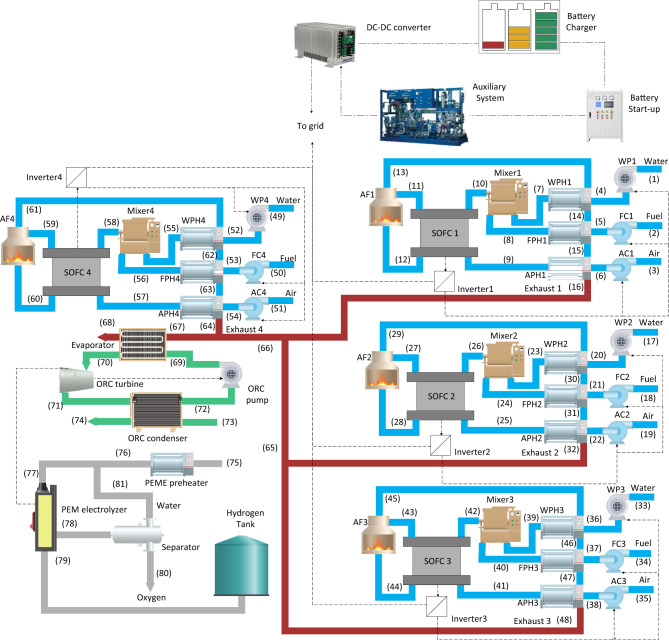
The detailed schematic of the dynamic cogeneration system to provide hydrogen and electricity as the charging station for the electric vehicles: The second scenario. (The figure has been obtained using Origin(Pro), Version *9.9*, 2022. OriginLab Corporation, Northampton, MA, USA, available at: https://www.originlab.com/).

Considering the storage unit, the working load of the SOFC stacks at night, once the power load is lower than the day, will be reduced. Thus, it is assumed that SOFC1, SOFC2, and SOFC3 will be working part-load (30%) from 10 pm to 6 am while SOFC4 will be always working full-load. However, considering the possibility of a sudden increase/decrease in the power demand, a battery is utilized to store the excessed electricity during the day and to generate the required electricity if there is a sudden high demand for power (see Fig. 2). For the proposed system, the energy and exergy analyses will be developed to calculate the thermodynamic characteristics in each state and to predict the overall efficiencies. The thermodynamic analysis is followed by an in-depth life-cycle assessment with eighteen meet-points and three end-points.

## Thermodynamic modeling

In this study, thermodynamic modeling has been done in MATLAB software, using the governing equations for the SOFC, ORC, and PEME units. Methane is the working fuel of the SOFC stack, which will be mixed with water vapor after being pre-heated and compressed. The output gases will be directed to the afterburner to produce the needed heat to be transferred to the pre-heaters. As the exhausts of the SOFC stacks (Exhaust 1, Exhaust 2, Exhaust 3, and Exhaust 4) are at high temperatures. An ORC, using R245fa as the working fluid, will re-use the output heat of the SOFC stacks to further improve the efficiency and provide the input electricity for the PEME. The authors have already presented the required governing equations to model the PEME in detail^25^, and they are not stated here to prevent repetition. The needed governing equations for the SOFC stacks and the ORC unit in addition to the exergy balance equations and the required expressions to calculate the overall energy and exergy efficiencies can be found in the Appendix.

### Battery modeling

**Figure 3:**
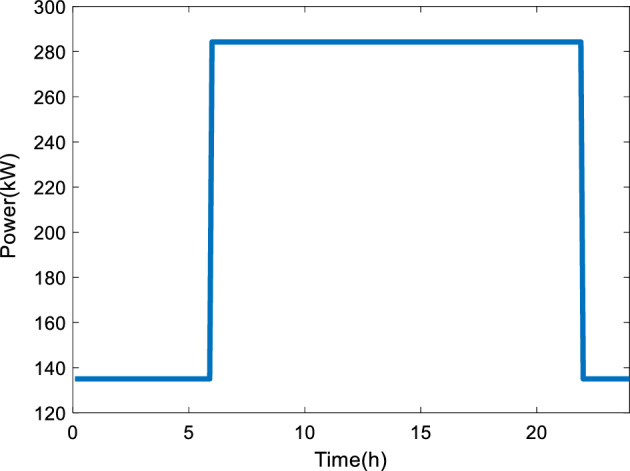
The profile of the power generation by the SOFC stacks, that is suggested in Fig. 2.

Based on Fig. 2, the difference between the electricity demand during the day and night should be calculated. The SOFC stacks will work partially by 30% power load from 22 to 6 h for eight hours. Figure 3 shows the considered profile of the power generation by the SOFC stacks based on Fig. 2.

A battery is being used to store the energy surplus during the night and can compensate for the deficit of electricity at peak hours. During the charging period of the battery, the charging current would be limited by a maximum charging current ${{I}_{C}}_{max}$. The theoretical value of the charging current is calculated as:

1 $${I}_{C}=\frac{{P}_{+}{\varepsilon }_{C}}{{V}_{B}}$$

where, ${P}_{+}$ (W)is the surplus power, ${\varepsilon }_{C}$ is the efficiency of the DC-DC converter, and ${V}_{B}$ (V) is the voltage across the battery. In this study, ${\varepsilon }_{C}$ is assumed to have a constant value of 95%.

During the discharging period, the battery would compensate for the lack of output power by the SOFC stacks to satisfy the power demand. In this case, the current is limited by a maximum discharging current of ${{I}_{D}}_{max}$, while the theoretical value can be calculated as follows:

2 $${I}_{D}=\frac{{P}_{-}}{{V}_{B}{\varepsilon }_{C}}$$

here, ${P}_{-}$ is the deficit of power output.

Having the charging and discharging currents, the state of charge (SOC) of the battery can be calculated. Equation (3) expresses the discharged capacity, ${C}_{D}$, during a period of $\Delta t$:

3 $${C}_{D}=\frac{{I}_{D}.\Delta t}{\alpha }$$

where $\alpha$ is the discharge efficiency given by the presented empirical Eq. (4):

4 $$\alpha =\frac{13.3\mathrm{ln}\left(\frac{{C}_{0}}{{I}_{D}}\right)+59.8}{100}$$

here, ${C}_{0}$ is the maximum capacity of the battery, hence $\alpha$ is limited to the values between zero and one.

Considering the negligible losses during the charging in comparison to the period of discharging, the changes in the capacity during $\Delta t$ can be presented as follows:

5 $$\Delta C={I}_{D}.\Delta t-{C}_{D}$$

Thus, the SOC status of the battery at time $t$ can be obtained by Eq. (6), considering the range of ($SO{C}_{min}, SO{C}_{max}$) for ${C}_{t}$:

6 $${C}_{t}={C}_{t-1}+\Delta C$$

## Results and discussion

The energy and exergy analyses of the suggested system have been performed using the REFPROP 9.0^26^ library in MATLAB software. The developed code for thermodynamic characterization considers the properties of each cycle using the input parameters given in Table 1. The output of this thermodynamic analysis will be the overall efficiencies in addition to the values of hydrogen production and output power. It should be noted that the authors have previously validated the SOFC unit, and PEME, which the validation figures are available on Refs.^25,27^, respectively, hence further explanation is not given in this study to prevent repetition.

**Table 1:** Input operating parameters to develop the energy and exergy characterizations.

Parameters	Value
SOFC	
Ambient pressure	1.013 bar
Ambient temperature	298.15 K
Active surface area	100 $c{m}^{2}$
Number of cells	600
Pressure ratio of the compressor	1.19
Pressure drop of fuel heat exchanger	0.02
Pressure drop of SOFC stack	0.02
Pressure drop of afterburner	0.03
Steam to carbon ratio	2.5
Inlet temperature	1000 K
ORC	
Pump efficiency	0.85
Turbine efficiency	0.85
PEM electrolyzer	
Working temperature	353 K
${E}_{act,a}$	76,000 kJ/mol
${ E}_{act,c}$	18,000 kJ/mol
${ \lambda }_{a}$	14
${ \lambda }_{c}$	10
*L*	${10}^{-6} (\mu m)$
${ J}_{a}^{ref}$	$1.7\times {10}^{5}$(A/${m}^{2}$)
${ J}_{c}^{ref}$	$4.6\times {10}^{3}$(A/${m}^{2}$)

In the ORC unit, the pump and turbine efficiencies are assumed to be constant. The goal of this study is to clarify the suitability of the suggested integrated system to be operated as a charging station for the EVs using energetic, exergetic, and environmental aspects. To improve the efficiency of the whole system, the ORC unit was utilized to observe the possibility of recovering the SOFC units’ exhaust heat. In other words, although the exact amounts of the recovered heat and the overall efficiencies are calculated, the main aim of this study was to analyze the integration of the ORC unit and monitor the possibility of waste heat recovery. In the long-term operation, if the utilized pump in the ORC unit experiences cavitation, an increase in friction loss, wear inclusions in the ORC working fluids, and a bad power supply, the efficiency will not be the same, hence the lower amount of recovered heat. However, the focus of this study is not to study the long-term operation of the system but rather to find a perfect balance between the integrated units and providing the environmental impacts. Energy and exergy analyses are also performed to help the decision makers about the suitability of the system in real applications.

### Thermodynamic analysis

The energetic and exergetic performances of the system have been modeled in each state point. Considering the illustrated schematics in Figs. 1 and 2, the thermodynamic characteristics of the charging station have been obtained in each state, which facilitates the characterization of the system. Using the thermodynamic properties given in Table 2, the output power of the SOFC stacks can be predicted at the current density of 0.7 A/cm^2^. It should be noted that the obtained exhaust gas temperature from the combustion chambers of each SOFC unit, will be cooled down by the water, fuel, and air pre-heaters to reduce the exhaust gas temperature from the combustion chamber from 1135 K to 441.5 K = 168.35 °C in each SOFC unit. In this regard, the overall exhaust gas temperature from all of the SOFC units (${T}_{67}$ = 441.5 K = 168.35 °C) is in the temperature range of 80 °C to 350 °C, which is considered suitable for heat recovery with an ORC unit^13^. The calculated enthalpy and temperature at the state point of “68”, which is the output of the SOFC stacks, enable the calculation of the recovered heat by the ORC unit, the generated electricity by the ORC unit and transferred to the PEME, and the respective value of the hydrogen production at the current density of 0.7 A/cm^2^. Table 3 demonstrates the thermodynamic characteristics of each state variable in the ORC and PEME units. It should be noted that the existing parameters in Table 3 and Table 4 are the temperature, T (K), pressure, P (bar), mass flow rate, $\dot{m}$ ($\frac{\mathrm{mol}}{\mathrm{s}}$), entropy, $s$ ($\frac{\mathrm{kJ}}{\mathrm{kg} \mathrm{K}}$), enthalpy, $h$ ($\frac{\mathrm{kJ}}{\mathrm{kg}}$), and exergy, ex (kW).

**Table 2:** The thermodynamic characteristics of the state variables of the SOFC stacks at the 0.7 A/cm^2^.

State	T (K)	P (bar)	$\dot{m}$ ($\frac{\mathrm{mol}}{\mathrm{s}}$)	$h$ ($\frac{\mathrm{kJ}}{\mathrm{kg}}$)	$s$ ($\frac{\mathrm{kJ}}{\mathrm{kg K}}$)	Ex (kW)	Mole fraction percentage			
							${H}_{2}O$	${H}_{2}$	${O}_{2}$	${N}_{2}$
1	298.15	1.013	0.5234	1890.1	6.6151	0	100	0	0	0
2	298.15	1.013	0.2094	− 74,909	186.2242	0	0	0	0	0
3	298.15	1.013	15.7123	8644	6.8588	0	0	0	21	79
4	298.15	1.2055	0.5234	1890.5	6.6153	0	100	0	0	0
5	314.8364	1.2055	0.2094	− 74,296	186.8807	0.0874	0	0	0	0
6	316.0224	1.2055	15.7123	9164	6.8588	6.5116	0	0	21	79
7	1000	1.1662	0.5234	71,890	168.1431	11.4312	100	0	0	0
8	1000	1.1662	0.2094	− 36,734	246.7817	4.2123	0	0	0	0
9	1000	1.1662	15.7123	30,316	234.4018	175.5608	0	0	21	79
10	1000	1.1662	0.7328	− 164,650	240.8343	189.1621	71.43	0	0	0
11	1077	1.1577	1.316	− 207,540	240.7294	63.2332	71.26	10.56	0	0
12	1077	1.1577	17.563	24,265	236.6783	198.7425	0	0	19.24	80.76
13	1135	1.123	16.445	7848	241.4281	240.4667	5.73	0	17.53	75.43
14	1079	1.1005	16.445	5918.5	239.793	213.3076	5.73	0	17.53	75.43
15	1066.5	1.07849	16.445	5490.2	239.543	207.4899	5.73	0	17.53	75.43
16	441.5	1.057	16.445	− 14,603	211.7672	13.2383	5.73	0	17.53	75.43
17	298.15	1.013	0.5234	1890.1	6.6151	0	100	0	0	0
18	298.15	1.013	0.2094	− 74,909	186.2242	0	0	0	0	0
19	298.15	1.013	15.7123	8644	6.8588	0	0	0	21	79
20	298.15	1.2055	0.5234	1890.5	6.6153	0	100	0	0	0
21	314.8364	1.2055	0.2094	− 74,296	186.8807	0.0874	0	0	0	0
22	316.0224	1.2055	15.7123	9164	6.8588	6.5116	0	0	21	79
23	1000	1.1662	0.5234	71,890	168.1431	11.4312	100	0	0	0
24	1000	1.1662	0.2094	− 36,734	246.7817	4.2123	0	0	0	0
25	1000	1.1662	15.7123	30,316	234.4018	175.5608	0	0	21	79
26	1000	1.1662	0.7328	− 164,650	240.8343	189.1621	71.43	0	0	0
27	1077	1.1577	1.316	− 207,540	240.7294	63.2332	71.26	10.56	0	0
28	1077	1.1577	17.563	24,265	236.6783	198.7425	0	0	19.24	80.76
29	1135	1.123	16.445	7848	241.4281	240.4667	5.73	0	17.53	75.43
30	1079	1.1005	16.445	5918.5	239.793	213.3076	5.73	0	17.53	75.43
31	1066.5	1.07849	16.445	5490.2	239.543	207.4899	5.73	0	17.53	75.43
32	441.5	1.057	16.445	− 14,603	211.7672	13.2383	5.73	0	17.53	75.43
33	298.15	1.013	0.5234	1890.1	6.6151	0	100	0	0	0
34	298.15	1.013	0.2094	− 74,909	186.2242	0	0	0	0	0
35	298.15	1.013	15.7123	8644	6.8588	0	0	0	21	79
36	298.15	1.2055	0.5234	1890.5	6.6153	0	100	0	0	0
37	314.8364	1.2055	0.2094	− 74,296	186.8807	0.0874	0	0	0	0
38	316.0224	1.2055	15.7123	9164	6.8588	6.5116	0	0	21	79
39	1000	1.1662	0.5234	71,890	168.1431	11.4312	100	0	0	0
40	1000	1.1662	0.2094	− 36,734	246.7817	4.2123	0	0	0	0
41	1000	1.1662	15.7123	30,316	234.4018	175.5608	0	0	21	79
42	1000	1.1662	0.7328	− 164,650	240.8343	189.1621	71.43	0	0	0
43	1077	1.1577	1.316	− 207,540	240.7294	63.2332	71.26	10.56	0	0
44	1077	1.1577	17.563	24,265	236.6783	198.7425	0	0	19.24	80.76
45	1135	1.123	16.445	7848	241.4281	240.4667	5.73	0	17.53	75.43
46	1079	1.1005	16.445	5918.5	239.793	213.3076	5.73	0	17.53	75.43
47	1066.5	1.07849	16.445	5490.2	239.543	207.4899	5.73	0	17.53	75.43
48	441.5	1.057	16.445	− 14,603	211.7672	13.2383	5.73	0	17.53	75.43
49	298.15	1.013	0.5234	1890.1	6.6151	0	100	0	0	0
50	298.15	1.013	0.2094	− 74,909	186.2242	0	0	0	0	0
51	298.15	1.013	15.7123	8644	6.8588	0	0	0	21	79
52	298.15	1.2055	0.5234	1890.5	6.6153	0	100	0	0	0
53	314.8364	1.2055	0.2094	− 74,296	186.8807	0.0874	0	0	0	0
54	316.0224	1.2055	15.7123	9164	6.8588	1374	0	0	21	79
55	1000	1.1662	0.5234	71,890	168.1431	11.4312	100	0	0	0
56	1000	1.1662	0.2094	− 36,734	246.7817	4.2123	0	0	0	0
57	1000	1.1662	15.7123	30,316	234.4018	175.5608	0	0	21	79
58	1000	1.1662	0.7328	− 164,650	240.8343	189.1621	71.43	0	0	0
59	1077	1.1577	1.316	− 207,540	240.7294	63.2332	71.26	10.56	0	0
60	1077	1.1577	17.563	24,265	236.6783	198.7425	0	0	19.24	80.76
61	1135	1.123	16.445	7848	241.4281	3.6831	5.73	0	17.53	75.43
62	1079	1.1005	16.445	5918.5	239.793	213.3076	5.73	0	17.53	75.43
63	1066.5	1.07849	16.445	5490.2	239.543	207.4899	5.73	0	17.53	75.43
64	441.5	1.057	16.445	− 14,603	211.7672	13.2383	5.73	0	17.53	75.43
65	441.5	1.057	32.89	− 14,603	211.7672	13,238	5.73	0	17.53	75.43
66	441.5	1.057	49.335	− 14,603	211.7672	13,238	5.73	0	17.53	75.43
67	441.5	1.057	65.78	− 14,603	211.7672	52.9532	5.73	0	17.53	75.43
68	301	1.03586	65.78	− 18,780	199.7856	3295	5.73	0	17.53	75.43

**Table 3:** The thermodynamic characteristics of the state variables in the ORC and PEME units.

State	T (K)	P (bar)	$\dot{m}$ ($\frac{\mathrm{mol}}{\mathrm{s}}$)	$h$ ($\frac{\mathrm{kJ}}{\mathrm{kg}}$)	$s$ ($\frac{\mathrm{kJ}}{\mathrm{kg K}}$)	ex (kW)
69	299.87	33.27	13.06	237.13	1.12	59.9
70	372	33.27	13.06	444.89	1.72	99.63
71	303.38	6.65	13.06	417.67	1.73	57.04
72	298.15	6.65	13.06	1.1199	1.12	56.98
73	298.15	1.01	0.09	1888.5	6.61	0
74	353	1.01	0.09	6019.6	19.33	0.19
75	353	1.01	0.11	6019.6	19.33	0.23
76	353	1.01	0.06	35,751.2	76.5	0.21
77	353	1.01	0.09	9516.7	112.61	19.96
78	353	1.01	0.04	50,617	105.09	0.17
79	353	1.01	0.02	6019.6	19.33	0.05

**Table 4:** The needed parameters of the battery to develop the dynamic analysis.

Parameters	Value
Voltage across cell	2.1 (V)
Maximum discharge rate	C/5
Maximum charge rate	C/10
Maximum cell capacity	50 (A.h.)
Lower capacity limit of the battery	20%
Maximum capacity limit of the battery	100%
The efficiency of the charge controller	98%

**Figure 4:**
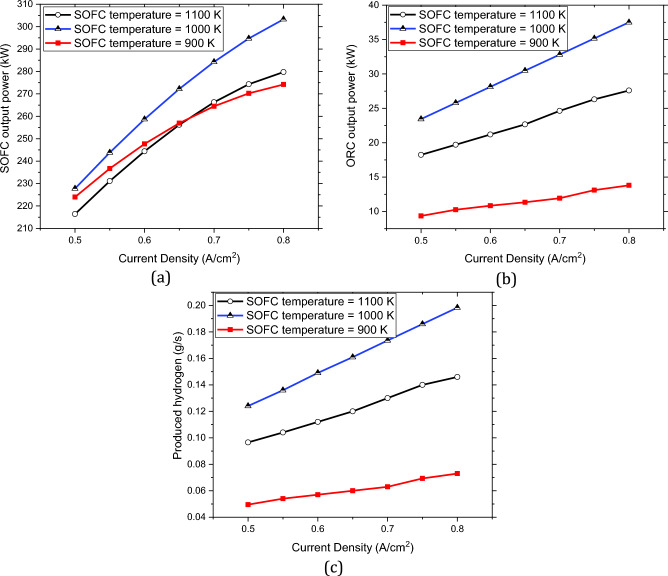
The impacts of the SOFC’s operating temperature and the current density on the outputs of the integrated system: (**a**) The variations in the output power of the SOFC stacks, (**b**) The variations in the output power of the ORC unit, (**c**) The variations in the hydrogen production.

With a similar approach to obtain the thermodynamic properties in different current densities of the SOFC stacks, the changes in the output parameters such as the output power by the SOFC stacks, the recovered heat by the ORC unit, and the produced hydrogen by the PEME can be obtained. Also, the variation in the operating temperature of the SOFC stacks has critical impacts on the overall performance of the charging station, hence the characteristics of the system should be evaluated at different operating temperatures and the current densities In this regard, Fig. 4 is provided to illustrate the impacts of these two parameters on the output power of the SOFC stack, the produced electricity by the ORC unit using the exhaust heat of the SOFC stacks, and the produced hydrogen using the output electricity of the ORC unit in different temperatures and the current densities of the SOFC stacks.

### Parametric study

**Figure 5:**
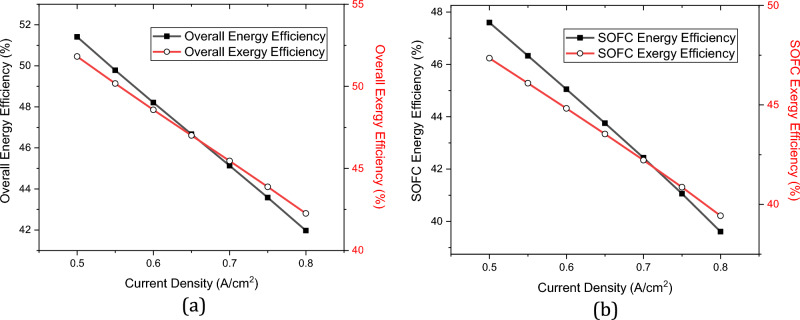
Efficiencies by the variation in the SOFC current density: (**a**) The changes in the overall efficiencies, (**b**) The changes in the SOFC efficiencies.

**Figure 6:**
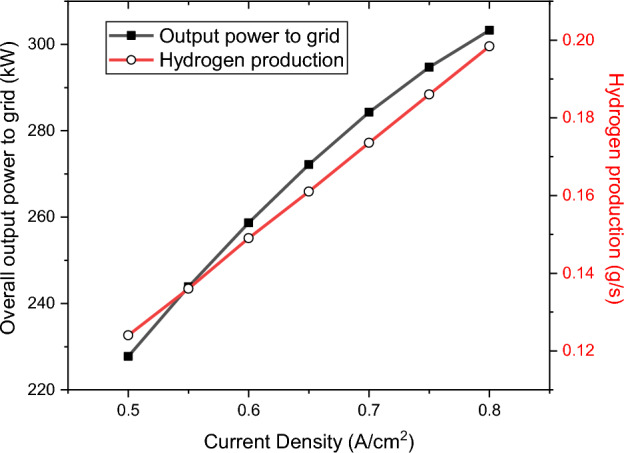
The values of the overall output power to the grid, and the hydrogen production by the variation in SOFC current density.

Once the output values are calculated, the determination of the efficiencies becomes feasible. Figure 5a shows the overall efficiencies of the integrated system by the variations in the current density of the SOFC stacks, while Fig. 5b illustrates those of the SOFC stack. Higher current densities of the SOFC stacks result in lower efficiencies in both the SOFC stacks and the whole system. Furthermore, Fig. 6 shows the impacts of SOFC current density on the output parameters of the charging station. The comparison of Figs. 5 and 6 shows although higher current densities result in lower efficiencies both in SOFC stacks and in the overall performance, it also results in higher production of hydrogen and electricity. Thus, a balance should be made to find the best current density to have the maximum possible output products with the highest possible efficiencies.

**Figure 7:**
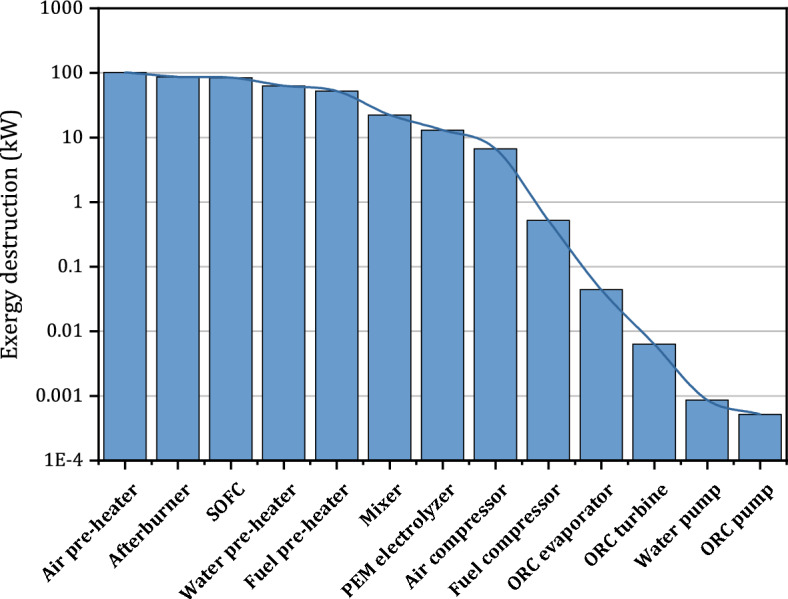
The exergy destruction values in each component of the integrated system.

In addition to the performance characterization of the system in different SOFC current densities, a detailed exergy analysis should be performed to obtain the required information about the efficiency of each component. The exergy destruction values can be calculated in each component to understand the efficiency of each component of the charging station. Figure 7 illustrates the exergy destruction values of the components that are utilized in the suggested integrated system. Air pre-heater has by far the highest exergy destruction followed by the afterburner of the SOFC stacks while the ORC pump has the least value among all the considered parameters.

**Figure 8:**
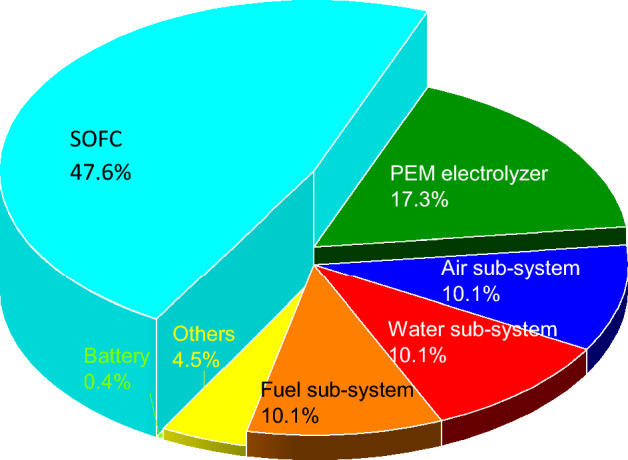
The estimated sizes of the components in the suggested integrated design for a charging station.

**Figure 9:**
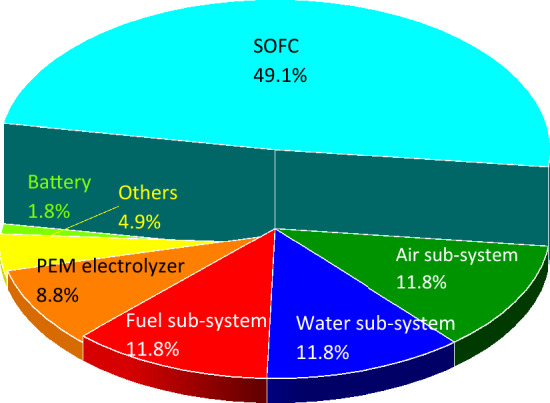
The estimated weights of the components in the suggested integrated design for a charging station.

Once the performance of the whole integrated system and each component of the system is revealed using thermodynamic modeling, the implementation challenges should be considered. The construction of a charging station using the suggested technologies demands space and specific materials to support the weight of the system. In this regard, size and weight analysis should be performed to provide the required infrastructure. Figure 8 shows the sizes of the main sub-sections of the system while Fig. 9 presents those of weights. As can be seen, the SOFC unit has the highest size and weight among the considered sub-sections followed by the PEME for the size and the air sub-system for the weight. The considered battery in the dynamic configuration, Fig. 2, has also a lower size and weight.

### Dynamic operation

**Figure 10:**
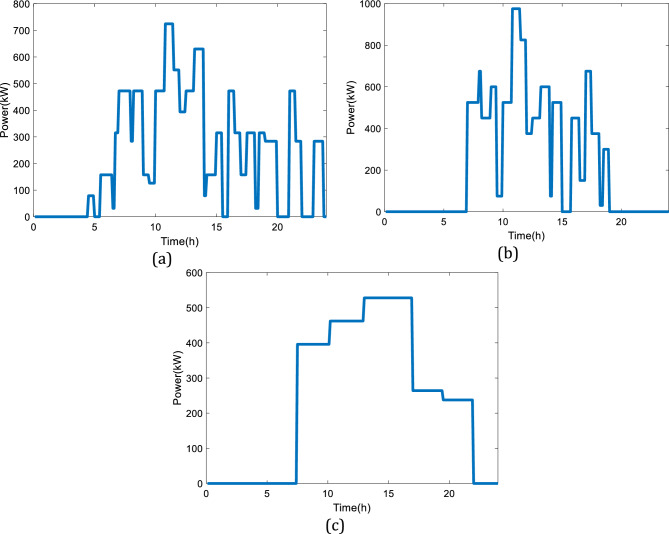
Three power loads of the charging station: (**a**) First profile, (**b**) Second profile, (**c**) Third profile.

Considering the given information about the dynamic configuration (see Fig. 2) in Sect. 2, a battery is combined with the initial design (see Fig. 1). Thus, the excessed electricity will be stored at low power demands, and the system will have a backup at high power loads. The input parameters to model the battery are given in Table 4, while Fig. 10 illustrates different selected power profiles of the charging stations to perform a dynamic study. The power load demand is inspired by Gilleran et al.^28^ with modifications to match the power generation.

**Figure 11:**
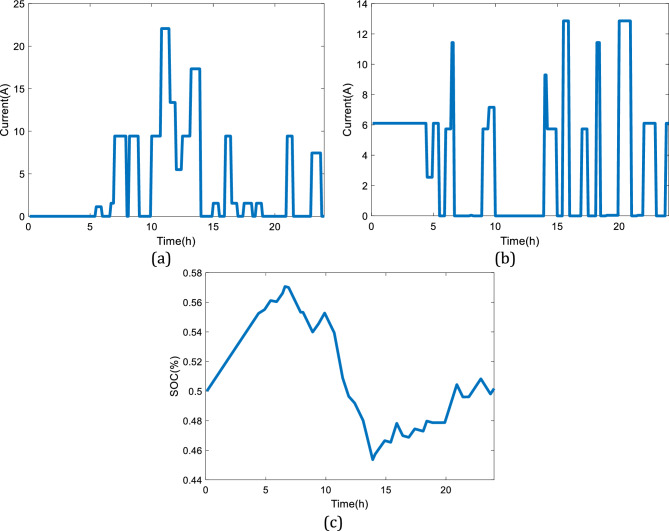
The simulation results considering the first power demand profile given in Fig. 10a: (**a**) Charging profile of the battery, (**b**) Discharging profile of the battery, (**c**) SOC of the battery.

Considering the presented power demand profile in Fig. 10a, the dynamic responses of the system are obtained (24 h). Figure 11a shows the charging profile of the considered battery while Fig. 11b presents the discharging profile. As can be seen, the system shows proper dynamic responses to the variations in the power demand profile given in Fig. 10a. Figure 11c illustrates the state of charge (SOC) of the battery by the changes in the power load during the day. It is noteworthy to mention that the design of the battery follows the maximum charging and discharging rate of C/10 and C/5, respectively. If the considered battery is smaller, hence different charging and discharging rate, then, the sudden changes in the power load cannot be responded to appropriately, and a safe region to protect the battery in real applications will not be provided. Meanwhile, the SOC of the considered battery as of Fig. 11c will be in the range of (40% to 60%), which will improve the dynamic response of the system and provide a safe operating condition for the battery.

**Figure 12:**
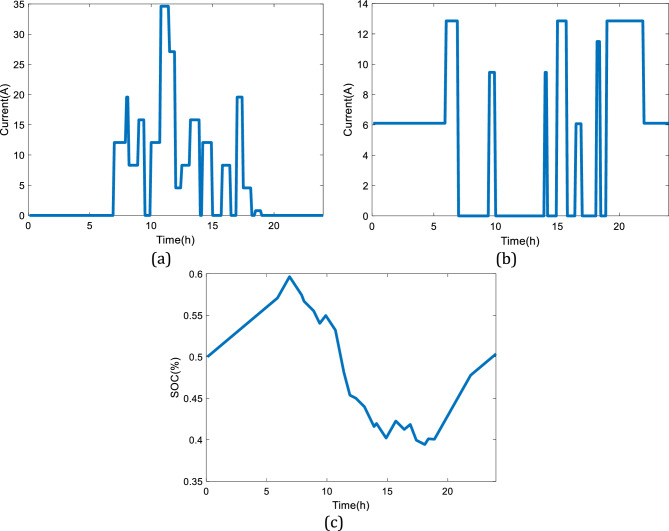
The simulation results considering the second power demand profile given in Fig. 10b: (**a**) Charging profile of the battery, (**b**) Discharging profile of the battery, (**c**) SOC of the battery.

A similar study has been performed considering the second power demand profile for the charging station based on Fig. 10b. The details of the dynamic responses of the battery to the variations in the power load of Fig. 10b are shown in Fig. 12. The second pattern presented in Fig. 12 experiences a high utilization level. When the variations in the power demand are drastic, the performance limitation of the battery will be the discharging current. According to Linden et al.^29^, the maximum discharging current should be C/5, hence the current will stay within the limit.

**Figure 13:**
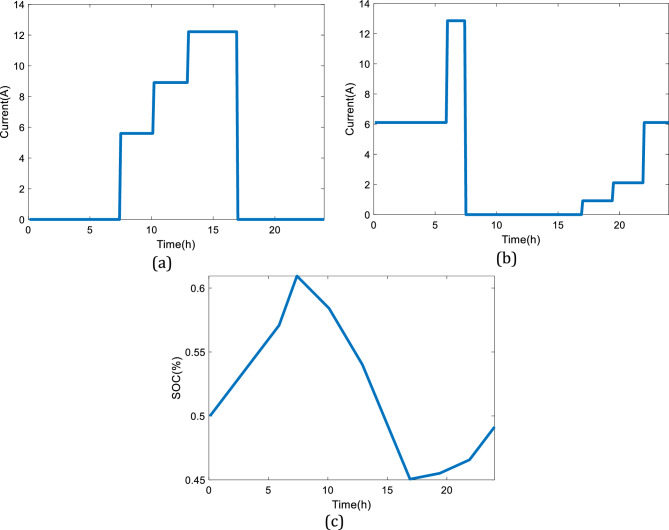
The simulation results considering the third power demand profile given in Fig. 10c: (**a**) Charging profile of the battery, (**b**) Discharging profile of the battery, (**c**) SOC of the battery.

Also, Fig. 13 demonstrates the charging, discharging, and SOC profiles of the battery with the variations in the power load given by Fig. 10c. The analyzed pattern for the third power profile has a high utilization level with a smooth variation in the power demand, which means having a durable deficit period during the day. In this case, the storage capacity of the battery is a critical factor to limit performance. Figure 13 shows that the utilized battery has a larger capacity than the produced electricity by the SOFC so that the SOC status is kept in a safe region during working hours.

### Life-cycle assessment (LCA)

Using the LCA as a method to obtain the environmental impacts of different technologies, the proposed design of the charging station has been evaluated. The LCA frameworks are based on ISO 14040^30^ and 14044^31^ using the ReCiPe 2016 v1.03 Midpoint (H) method^32^. The openLCA software, which is an open-source software to perform LCA, has been utilized to perform the simulations. The dedicated database “ecoinvent”^33^ enjoys a transparent unit-process LCI database. Table 5 presents the needed input materials to build up the suggested integrated design of the charging station without the consideration of the electrolyzer unit.

**Table 5:** The input information to develop LCA using Methane as the SOFC’s fuel and R134a as ORC’s working fluid^34–36^.

Inputs	Values
Air	1638 kg
Alumina	10 kg
Aluminum oxide sealer	84 kg
Anode graphite for Li-Ion battery	250 kg
Battery separator	250 kg
Cerium Lanthanum Yttrium Carbonate Phosphor	29.4 kg
Chlorine	163.2 kg
Ethanol	44.88 kg
Ethylene Glycol Polyol	2.88 kg
Heat by the onsite boiler	44.28 GJ
Heating and sanitary equipment	0.48 items
Inverter	40 items
Iron-Nickel–Chromium alloy	40 kg
Lanthanum oxide	270.24 kg
Li-Ion battery cell	250 kg
${\mathrm{LiMn}}_{2}{\mathrm{O}}_{4}$ cathode for Li-Ion battery	250 kg
Medium voltage electricity	1.07 MWh
Methane	48.24 kg
Nickel	0.024 kg
Nickel, 99.5%	4 kg
Nickel, Ni 2.5E + 0%, in mixed ore	18.72 kg
Nitric acid, without water, in 50% solution state	1367.04 kg
ORC for heat and power co-generation	1 unit
Polyvinyl butyral (PVB)	7.44 kg
Sheet rolling, chromium steel	328 kg
Sheet rolling, steel	1880 kg
SOFC maintenance	4 units
SOFC stacks	4 units
Spray drying process	57 GJ
Steel reinforcement bare; Blast furnace route	1880 kg
Steel, chromium steel 18/8	488 kg
Transport freight, sea, container ship	2.0544E4 t.km
Transport, freight train	3756.8 t.km
Transport, freight, light commercial vehicle	100 t.km
Transport, freight, lorry 16–32 metric ton, EURO6	512 t.km
Transport, lorry > 28t, fleet average—US	256 t.km
Transport, lorry 16-32t, EURO3—US	576 t.km
Urea, as N	672 kg
Water for manufacturing	12.48 m^3^
Water, deionized	236.16 kg
Zirconium Chloride powder	199.68 kg
Zirconium oxide	18.72 kg

This study also evaluates the environmental impacts of different existing devices to act as the electrolyzer unit in the proposed charging station. So far, the most commercialized electrolyzers are the Solid Oxide Electrolyzer (SOE), Proton Exchange Membrane Electrolyzer (PEME), and Alkaline Electrolyzer (ALE). The needed input materials to produce 5 m^2^ of single repeating unit of ALE, PEME, and SOE, are demonstrated in Tables 6, 7, and 8, respectively.

**Table 6:** The required input parameters to produce 5 m^2^ of single repeating unit of Alkaline electrolyzer (ALE)^37,38^.

Input	Value	Input	Value
Manufacture waste (g)	1580	Polyphenylene sulfide (g)	9720
Nickel (g)	34,735	Stainless steel (g)	23,280
Nickel plate (g)	34,735	Zirfon (g)	610
Nickel sulfide coating (g)	200

**Table 7:** The required input parameters to produce 5 m^2^ of single repeating unit of proton exchange membrane electrolyzer (PEME)^39–43^.

Input	Value	Input	Value
Carbon paper (g)	990	Platinum coating (g)	70
Ink materials (g)	1615	Platinum sputter (g)	105
Iridium (g)	65	Recycled noble materials (g)	235
Manufacture waste (g)	2395	Rubber gasket (g)	105
Nafion 115 (g)	835	Stainless steel (g)	11,850
Other metals (g)	4845	Titanium felt (g)	1130
Platinum (g)	40	Titanium plate (g)	47,315

**Table 8:** The required input parameters to produce 5 m^2^ of the single repeating unit of solid oxide electrolysis cell (SOEC)^44,45^.

Input	Value	Input	Value
Aluminum oxide (Al_2_O_3_) (g)	195	Nickel oxide—8 mol% yttria-stabilized zirconia (NiO-8YSZ) (g)	470
Ferritic stainless steel (g)	20,850	Other metals (g)	68,045
Glass–ceramic (g)	100	Spinal protection layers of (${\mathrm{Mn}}_{1.5}{\mathrm{CO}}_{1.5}{\mathrm{O}}_{4}$) on ferritic stainless steel (g)	160
Lanthanum Strontium Cobalt Ferrite (LSCF) (g)	360	Stainless steel (g)	77,100
Manufacture waste (g)	3,315	Tape casting slurry (g)	11,130
Ni/Gadolinium-Doped Ceria (CGO10) (g)	375	Yttria-stabilized zirconia (YSZ) (g)	280
Nickel oxide—3 mol% yttria-stabilized zirconia (NiO-3YSZ) (g)	665	Yttrium oxide (${Y}_{2}{O}_{3}$) (g)	85

**Figure 14:**
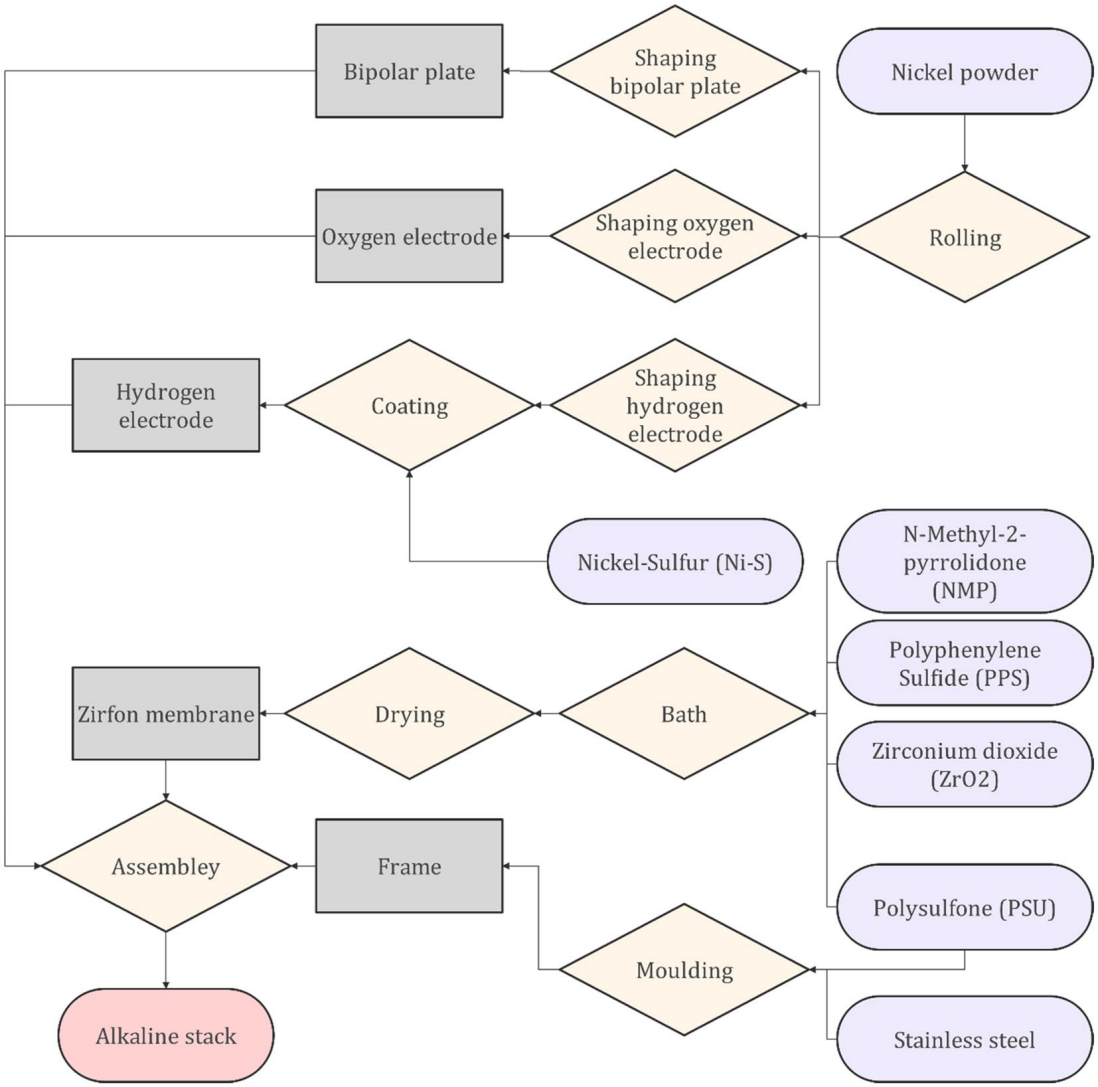
The procedure to manufacture the Alkaline electrolyzer that has been used in this study.

In addition to the needed materials to produce the electrolyzer unit, different types of processes are required for each electrolyzer type. In this regard, the overall environmental impact will change if the type of electrolyzer changes. Figure 14 presents the procedure to manufacture the ALE to be combined into the proposed integrated design. In the first step, the Nickel powder should go through the rolling process followed by shaping to form the bipolar plates and electrodes. The Zirfon membrane can be also produced using the bathing and drying processes using N-Methyl-2-pyrrolidone (NMP), Polyphenylene Sulfide (PPS), Zirconium dioxide (Zr ${\mathrm{O}}_{2}$), and Polysulfone (PSU) as the input materials. Additionally, the frames, which are made of stainless steel, will be manufactured by molding.

**Figure 15:**
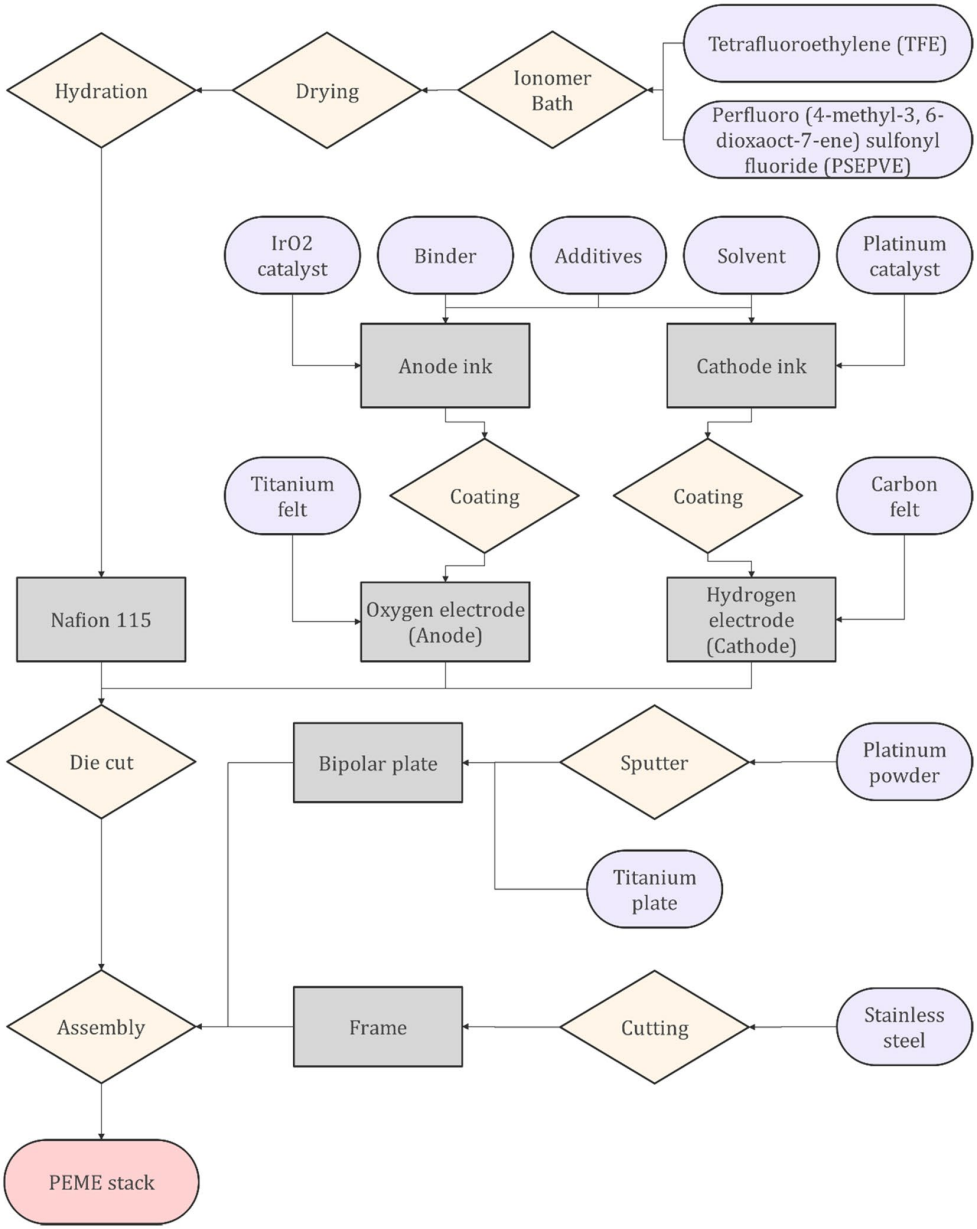
The procedure to produce the proton exchange membrane electrolyzer that has been used in this study.

Similarly, a membrane, electrodes, bipolar plates, and frames should be manufactured for the PEME. The bipolar plate is a combination of Platinum and Titanium using sputtering, while the frame will be produced by cutting the stainless steel. To generate the electrodes, binders, additives, and solvents will be used with Iridium oxide, and Platinum catalysts to form the required ink materials. After coating, Titanium, and carbon felt will be used to form the corresponding oxygen and hydrogen electrodes. Nafion 115 membrane will be also produced after passing through different processes such as bathing, drying, and hydration. Figure 15 presents the procedure to manufacture the PEME to be integrated into the suggested integrated design.

**Figure 16:**
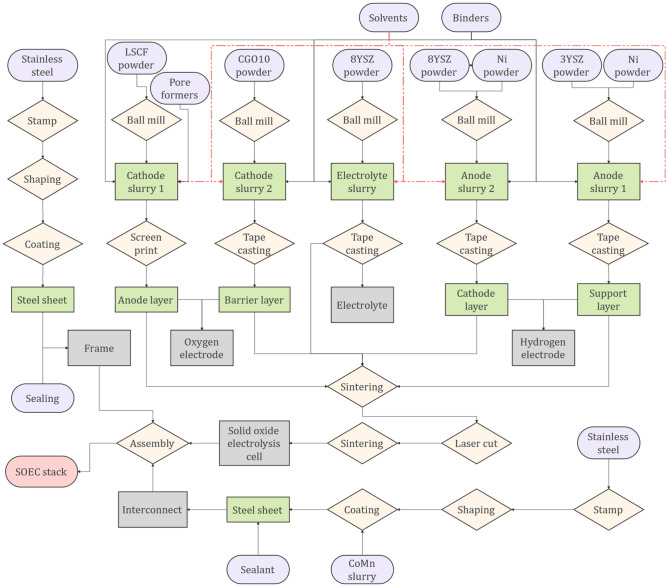
The procedure to produce a solid oxide electrolyzer that has been used in this study.

Figure 16 also illustrates the required manufacturing processes and materials for the SOE. As can be seen, the interconnects are being produced using the stamping, shaping, and coating of the stainless steel, similar to the frames. Screen printing is being utilized to form the oxygen electrode while the hydrogen electrode and electrolyte only require tape casting and ball milling. After the production of the electrodes and the electrolyte, the sintering and laser cutting processes will be used to form a solid oxide electrolysis cell (SOEC), which will be later assembled to the frame, and interconnect to make a SOEC stack.

Once the modeling of the input materials has been done in the OpenLCA software, the environmental impacts of the system in Fig. 2 can be obtained considering three different technologies to act as the electrolyzer unit, namely, SOEC, PEMEC, and AEC. Table 9 provides the LCA output results concerning different types of electrolyzers based on ReCiPe 2016 v1.03 Midpoint (H) for the suggested design of the 284.27 kWh. Results indicate the suitability of the PEMEC considering the environmental aspects while voting against using SOEC from this viewpoint. Furthermore, Table 10 presents the changes in the 18 environmental parameters to the variations in input fuel of the SOFC stacks. Results indicate that hydrogen has by far the least adverse influence on the environment followed by the bio-methanol to act as the fuel source for the four SOFC stacks.

**Table 9:** The comparison between the environmental impacts of different types of electrolyzer technologies if they are being integrated into the suggested design of the charging station is based on ReCiPe 2016 v1.03 Midpoint (H) for the proposed design of the 284.27 kWh.

Impact category	Unit	SOEC	PEMEC	AEC
Fine particulate matter formation (FPMF)	kg PM2.5-eq	1.65E+03	1.43E+03	1.52E+03
Fossil resource scarcity (FRS)	kg oil-eq	1.33E+05	1.15E+05	1.22E+05
Freshwater ecotoxicity (FE)	kg 1,4-DCB eq	1.49E+05	1.29E+05	1.37E+05
Freshwater eutrophication (FEu)	kg P-eq	3.37E+02	2.91E+02	3.10E+02
Global warming (GW)	kg CO_2_ eq	5.17E+05	4.47E+05	4.76E+05
Human carcinogenic toxicity (HCT)	kg 1,4-DCB eq	1.58E+06	1.23E+05	1.45E+05
Human non-carcinogenic toxicity (HnCT)	kg 1,4-DCB eq	1.42E+05	1.37E+06	1.31E+06
Ionizing radiation (IR)	kBq Co-60-eq	2.93E+04	2.53E+04	2.70E+04
Land use (LU)	m^2^a crop-eq	1.35E+04	1.17E+04	1.24E+04
Marine ecotoxicity (ME)	kg 1,4-DCB eq	1.91E+05	1.65E+05	1.76E+05
Marine eutrophication (MEu)	kg N-eq	1.86E+01	1.61E+01	1.71E+01
Mineral resource scarcity (MRS)	kg Cu eq	1.86E+04	1.61E+04	1.71E+04
Ozone formation, Human health (OFH)	kg NO_x_ eq	1.48E+03	1.28E+03	1.36E+03
Ozone formation, Terrestrial ecosystems (OFT)	kg NOx-eq	1.54E+03	1.33E+03	1.42E+03
Stratospheric ozone depletion (SOD)	kg CFC11-eq	1.04E+00	9.04E−01	9.58E−01
Terrestrial acidification (TA)	kg SO2-eq	3.77E+03	3.26E+3	3.47E+00
Terrestrial ecotoxicity (TE)	kg 1,4-DCB eq	7.71E+06	6.67E+06	7.10E+06
Water consumption (WC)	m^3^	4.71E+03	4.07E+03	4.34E+03

**Table 10:** The comparison between the environmental impacts of different types of SOFC fuel for the proposed design of the 284.27kWh charging station based on ReCiPe 2016 v1.03 Midpoint (H).

Impact category	Unit	Methane	Bio-methanol	Natural gas	Biogas	Hydrogen
FPMF	kg PM2.5-eq	1.43E+03	8.32E+03	3.67E+02	9.27E+02	2.40E+02
FRS	kg oil-eq	1.15E+05	2.46E+04	5.91E+04	1.26E+04	3.32E+04
FE	kg 1,4-DCB eq	1.29E+05	2.75E+04	6.63E+04	1.42E+04	3.72E+04
FEu	kg P-eq	2.91E+02	4.73E+02	5.74E+01	4.61E+01	6.56E+01
GW	kg CO_2_ eq	4.47E+05	7.23E+04	2.16E+05	1.71E+05	5.87E+04
HCT	kg 1,4-DCB eq	1.23E+05	1.99E+04	5.95E+04	4.71E+04	1.61E+04
HnCT	kg 1,4-DCB eq	1.37E+06	2.21E+05	6.62E+05	5.25E+05	1.80E+05
IR	kBq Co-60-eq	2.53E+04	4.09E+03	1.23E+04	9.67E+03	3.32E+03
LU	m^2^a crop-eq	1.17E+04	1.89E+03	5.66E+03	4.48E+03	1.53E+03
ME	kg 1,4-DCB eq	1.65E+05	2.67E+04	7.98E+04	6.31E+04	2.17E+04
MEu	kg N-eq	1.61E+01	2.62E+01	3.18E+00	2.55E+00	3.64E+00
MRS	kg Cu eq	1.61E+04	2.62E+04	3.18E+03	2.55E+03	3.64E+03
OFH	kg NO_x_ eq	1.28E+03	3.39E+02	8.08E+02	1.97E+02	2.37E+02
OFT	kg NOx-eq	1.33E+03	1.33E+03	8.37E+02	2.05E+02	2.45E+02
SOD	kg CFC11-eq	9.04E−01	2.40E−01	5.72E−01	1.40E−01	1.68E−01
TA	kg SO2-eq	3.26E+3	8.62E+02	2.05E+03	5.03E+02	6.03E+02
TE	kg 1,4-DCB eq	6.67E+06	1.76E+06	4.20E+06	1.03E+06	1.24E+06
WC	m^3^	4.07E+03	1.08E+03	2.57E+03	6.27E+02	7.53E+02

This study also characterizes the environmental impacts of different ORC’s working fluids. Table 11 presents a comparison between R134a, R227ea, and R152a considering their environmental impacts. Results show that once R152a has been used as the ORC’s working fluid, the integrated design of the system shown in Fig. 2 has the lowest production of carbon dioxide by 4.02E+05 (kg CO_2_ eq), while that of R227ea leads to the highest amount by the generation of 6.19E+05 (kg CO_2_ eq) carbon dioxide.

**Table 11:** The comparison between the environmental impacts of different types of ORC’s working fluid for the proposed design of the 284.27kWh charging station based on ReCiPe 2016 v1.03 Midpoint (H).

Impact category	Unit	R134a	R227ea	R152a
FPMF	kg PM2.5-eq	1.43E+03	1.98E+03	1.28E+03
FRS	kg oil-eq	1.15E+05	1.59E+05	1.04E+05
FE	kg 1,4-DCB eq	1.29E+05	1.78E+05	1.16E+05
FEu	kg P-eq	2.91E+02	4.03E+02	2.62E+02
GW	kg CO_2_ eq	4.47E+05	6.19E+05	4.02E+05
HCT	kg 1,4-DCB eq	1.23E+05	1.71E+05	1.11E+05
HnCT	kg 1,4-DCB eq	1.37E+06	1.90E+06	1.23E+06
IR	kBq Co-60-eq	2.53E+04	3.51E+04	2.28E+04
LU	m^2^a crop-eq	1.17E+04	1.62E+04	1.05E+04
ME	kg 1,4-DCB eq	1.65E+05	2.29E+05	1.49E+05
MEu	kg N-eq	1.61E+01	2.23E+01	1.45E+01
MRS	kg Cu eq	1.61E+04	2.23E+04	1.45E+04
OFH	kg NO_x_ eq	1.28E+03	1.77E+03	1.15E+03
OFT	kg NOx-eq	1.33E+03	1.84E+03	1.20E+03
SOD	kg CFC11-eq	9.04E−01	1.25E+00	8.14E-01
TA	kg SO2-eq	3.26E+03	4.52E+03	2.93E+03
TE	kg 1,4-DCB eq	6.67E+06	9.26E+06	6.00E+06
WC	m^3^	4.07E+03	5.63E+03	3.66E+03

**Figure 17:**
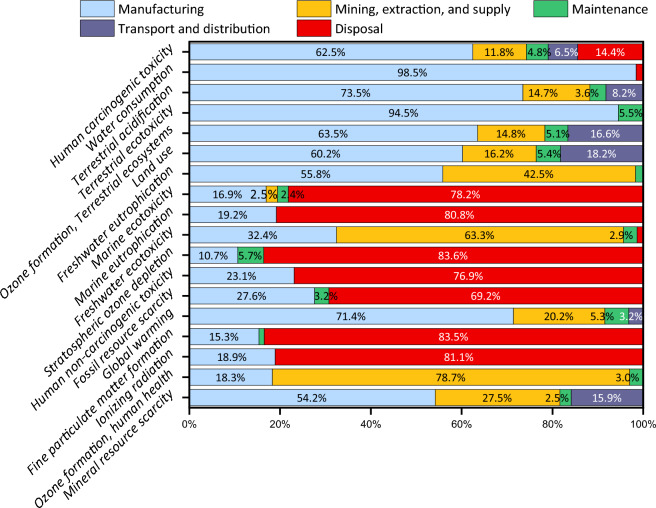
The shares of different processes on the environment during the operation of the 284.27 kWh charging station with PEMEC as the electrolyzer.

Another characterization of the system has been done once Methane, R134a, and PEME have been chosen as the SOFC’s input fuel, ORC’s working fluid, and the electrolyzer unit using five main categories (see Fig. 17). Based on the obtained results, manufacturing plays a critical role on the HCT, WC, TA, TE, OFT, LU, and FEu, while the disposal is the main driver of the ME, MEu, SOD, HnCT, FRS, FPMF, and IR.

## Conclusion

This study evaluated the possibility of using SOFC technology to act as the provider of electricity for electric vehicles in a charging station. The outcomes of this study facilitate the transition from fossil fuel-based technologies to environmentally friendly alternatives. In the steady-state condition, the waste heat of the SOFC stacks was re-used by an ORC unit and used as an input for the electrolyzer unit. The thermodynamic analysis of the system shown in Fig. 1, revealed that the overall energy and exergy efficiency of the system at the operating current density of 0.7 A/${\mathrm{cm}}^{2}$ are 60.84% and 60.67%, respectively, with the corresponding power and hydrogen production of 284.27 kWh and 0.17 g/s. The result indicated that higher current densities of the SOFC stacks lead to lower efficiencies both in the fuel cell and the integrated system while increasing hydrogen production and electricity generation. In this regard, an optimum operating current density should be found to reach the highest efficiency in the highest production of hydrogen and electricity. The results of the exergy destruction study revealed that the air preheater has by far the highest value followed by the afterburner while the ORC pump has the lowest exergy destruction. The obtained results indicated that the current suggested system with ORC exhaust heat recovery can act as the charging station for EVs. The overall energy efficiency of fossil fuel-based power generation systems is around 15% to 45%, however, the current system benefits from the energy efficiency of 60.84%.

In the dynamic configuration, Fig. 2, the ${\mathrm{LiMn}}_{2}{\mathrm{O}}_{4}$ lithium-ion battery was combined into the system to facilitate the partial operation (30%) of the SOFC1, SOFC2, and SOFC3 from 10 pm to 6 am. Once the power demand was low, the battery could store the electricity and act as a backup in critical conditions. The dynamic response of the integrated system with batteries demonstrated the suitability and stability of the suggested system in three different arbitrary power loads during the day. The size and weight study also revealed that the battery benefits from the lowest volume and weight in comparison to the other components of the system. Among the considered components of the system, the SOFC unit and the PEME have by far the highest volume. To obtain the environmental impacts of the integrated system the LCA model was developed for the 284.27 kWh system and the results showed that the system leads to global warming (kg ${\mathrm{CO}}_{2}$ eq) of 5.17E+05, 4.47E+05, and 5.17E+05 using Solid Oxide Electrolyzer (SOE), Proton Exchange Membrane Electrolyzer (PEME), and Alkaline Electrolyzer (ALE), respectively. A comparison was also made using different types of fuel for the SOFC stacks and the results indicated that global warming (kg ${\mathrm{CO}}_{2}$ eq) of 4.47E+05, 7.23E+04, 2.16E+05, 1.71E+05, and 5.87E+04 when methane, bio-methanol, natural gas, biogas, and hydrogen are being used, respectively. A comparison between the environmental impacts of different types of ORC’s working fluids also suggested against the usage of R227ea while R152a showed promising results to be used in the system.

Although the outputs of this study have covered many important aspects, further investigations can be done as a topic for future studies as follows:

- One of the critical aspects of designing a power generation system using SOFC stacks is the slow dynamic response in this type of fuel cell. Switching the SOFC from full load to partial load may take several hours, and during this time, the SOFC is not able to provide power. In this regard, a study on the thermal hysteresis of SOFC stacks during SOFC load switching is of interest for future studies.
- The aims of this study were mainly on suggesting an efficient power-generating system for EVs and evaluating its performance accounting for the energetic, exergetic, and LCA aspects. The goal of this study was to provide technical engineering information on how the system would be in reality and what are the advantages/disadvantages of implementing this system rather than considering the business aspects. Thus, research on the cost analysis for the suggested system can be an interesting topic for future studies.
- In this study, it was concluded that higher current density of the SOFC stacks leads to lower efficiencies of these stacks, hence reducing overall performance. This study has only considered the impacts of the current density, which is common to regulate the output power of the SOFC stacks. However, other factors such as fuel utilization, air excess ratio, etc. can be interesting to study for future research.

## Supplementary Information

Supplementary Information.

## Data Availability

Available upon formal request from the corresponding author.
