# Supplementary material for: The thermodynamic and life-cycle assessments of a novel charging station for electric vehicles in dynamic and steady-state conditions
Source: Sci Rep. 2023 Jul 10;13:11159. doi: 10.1038/s41598-023-38387-0 (PMC10333262; doi:10.1038/s41598-023-38387-0)
Supplement: Supplementary file 1 — Supplementary Information. [file 41598_2023_38387_MOESM1_ESM.docx]

**Appendix**

## Solid Oxide Fuel Cell (SOFC)

The utilized SOFC stacks in ‎Fig. 1 are validated with the published experimental and simulation results in [28], [29]. The main concepts behind the chemical reactions that will happen in a SOFC are as follows:

| $CH_{4}+H_{2}O\to3H_{2}+CO$ | (7) |
| --- | --- |
| $H_{2}+\frac{1}{2}O_{2}\to H_{2}O+e^{-}$ | (8) |

Leading to the generated power given by Eq. (9):

| $W_{SOFC}=iA_{cell}N_{cell}V_{cell}$ | (9) |
| --- | --- |

here, i (A/$cm^{2}$) is the current density, $A_{cell}$ ($cm^{2}$) is the cell’s active area, $N_{cell}$ is the number of cells in each stack, and $V_{cell}$ (V) is the cell voltage as follows:

| $V_{cell}=V_{N}-V_{act}-V_{ohm}-V_{conc}$ | (10) |
| --- | --- |

where, $V_{N}$ (V) (see Eq. (11)), $V_{act}$ (V), $V_{ohm}$ (V), and $V_{conc}$ (V) are the Nernst, activation, Ohmic, and concentration voltages, respectively.

| $V_{N}=\left( -\frac{\Delta g_{s}}{nF} \right)-\frac{R_{u}T_{cell}}{nF}\ln\left( \frac{P_{H_{2}O}}{P_{H_{2}}\sqrt{P_{O_{2}}}} \right)$ | (11) |
| --- | --- |

In Eq. (11), $F$ (C/mol) is the Faraday constant, $R_{u}$ is the universal gas constant, $T_{cell}$ [K] is the cell’s operating temperature, $\Delta_{g_{s}}$ [kJ/kmol] is the changes in the molar Gibbs free energy, $n$ is the number of transferred electrons in the reaction, and $P_{i}$ (bar) is the partial pressure of each gas. The overall energy and exergy efficiencies for SOFC stacks are given by Eqs. (12) and (13):

| $\eta_{en}=\frac{W_{net}}{\dot{m}_{fuel}LHV_{fuel}}$ | (12) |
| --- | --- |
| $\eta_{ex}=\frac{W_{net}}{\dot{m}_{fuel}ex_{fuel}}$ | (13) |

here, $W_{net}$ (kW) is the net produced power by a SOFC stack, $\dot{m}$ (mol/s) is the methane’s molar flow rate, $ex_{fuel}$ (kJ/kmol) is the standard chemical exergy of the fuel, and $LHV_{fuel}$ (kJ/mol) is the Low Heating Value of methane.

## Organic Rankine cycle (ORC)

The utilized working fluid for the considered ORC is R245fa to ameliorate the turbine’s lifetime. In Evaporator, the working fluid reaches saturation as follows:

| $Q_{in,ORC}=\dot{m}_{67}\left( h_{68}-h_{67} \right)=\dot{m}_{69}(h_{70}-h_{69})$ | (14) |
| --- | --- |

where, $Q_{in,ORC}$ (W) is the exhaust heat by the GT given to the ORC to generate the electricity at the ORC turbine as follows:

| $W_{ORC,turbine}=\dot{m}_{70}(h_{70}-h_{71})$ | (15) |
| --- | --- |

The required power by the ORC pump is also as follows:

| $W_{ORC,pump}=\dot{m}_{72}(h_{69}-h_{72})$ | (16) |
| --- | --- |

Energy and exergy efficiencies of the considered ORC are demonstrated by Eqs. (17) and (18) as follows:

| $\eta_{en}=\frac{W_{ORC,turbine}-W_{ORC,pump}}{Q_{in,ORC}}$ | (17) |
| --- | --- |
| $\eta_{ex}=\frac{W_{ORC,turbine}-W_{ORC,pump}}{{Ex}_{in,ORC}}$ | (18) |

## Exergy analysis

The exergy value is a summation of physical and chemical exergies in each thermodynamic state. At the inlet flow of units, the physical exergy is zero, hence the exergy values can be only determined by knowing the chemical exergy values. Afterward, following the exergy balance equations given in ‎Table 12, the required exergy values to determine the exergy destructions in each component in addition to the overall exergy efficiency can be calculated.

After obtaining exergy values in each thermodynamic state, the overall energy and exergy efficiencies of the integrated system can be calculated by the following equations:

| $\eta_{en}=\frac{W_{net, SOFC, 1,2,3,4}+\dot{m}_{H_{2},out}h_{H_{2},out}}{\dot{Q}_{net,in}}$ | (19) |
| --- | --- |
| $\eta_{ex}=\frac{W_{net, SOFC, 1,2,3,4}+\dot{m}_{H_{2},out}{ex}_{H_{2},out}}{\dot{m}_{fuel}ex_{fuel}}$ | (20) |

| 1. Exergy balance equations for the current suggested system. | |
| --- | --- |
| Component | Exergy balance equation |
| **SOFC 1** |  |
| SOFC stack | $\dot{E}x_{10}^{ch}+\dot{E}x_{10}^{ph}+\dot{E}x_{9}^{ch}+\dot{E}x_{9}^{ph}=\dot{E}x_{11}^{ch}+\dot{E}x_{11}^{ph}+\dot{E}x_{12}^{ch}+\dot{E}x_{12}^{ph}+\dot{E}x_{D, SOFC}$ |
| Water pump | $\dot{E}x_{1}+W_{WP}=\dot{E}x_{4}+\dot{E}x_{D,WP}$ |
| Fuel compressor | $\dot{E}x_{2}+W_{comp}=\dot{E}x_{5}+\dot{E}x_{D,comp}$ |
| Air compressor | $\dot{E}x_{3}+W_{comp}=\dot{E}x_{6}+\dot{E}x_{D,comp}$ |
| Water preheater | $\dot{E}x_{13}+\dot{E}x_{4}=\dot{E}x_{7}+\dot{E}x_{14}+\dot{E}x_{D,WPH}$ |
| Fuel preheater | $\dot{E}x_{14}+\dot{E}x_{5}=\dot{E}x_{8}+\dot{E}x_{15}+\dot{E}x_{D,FPH}$ |
| Air preheater | $\dot{E}x_{15}+\dot{E}x_{6}=\dot{E}x_{9}+\dot{E}x_{16}+\dot{E}x_{D,APH}$ |
| Fuel mixer | $\dot{E}x_{7}+\dot{E}x_{8}=\dot{E}x_{10}+\dot{E}x_{D,mixer}$ |
| Afterburner | $\dot{E}x_{11}+\dot{E}x_{12}=\dot{E}x_{13}+\dot{E}x_{D,afterburner}$ |
| **SOFC 2** |  |
| SOFC stack | $\dot{E}x_{26}^{ch}+\dot{E}x_{26}^{ph}+\dot{E}x_{25}^{ch}+\dot{E}x_{25}^{ph}=\dot{E}x_{27}^{ch}+\dot{E}x_{27}^{ph}+\dot{E}x_{28}^{ch}+\dot{E}x_{28}^{ph}+\dot{E}x_{D, SOFC}$ |
| Water pump | $\dot{E}x_{17}+W_{WP}=\dot{E}x_{20}+\dot{E}x_{D,WP}$ |
| Fuel compressor | $\dot{E}x_{18}+W_{comp}=\dot{E}x_{21}+\dot{E}x_{D,comp}$ |
| Air compressor | $\dot{E}x_{19}+W_{comp}=\dot{E}x_{22}+\dot{E}x_{D,comp}$ |
| Water preheater | $\dot{E}x_{29}+\dot{E}x_{20}=\dot{E}x_{23}+\dot{E}x_{30}+\dot{E}x_{D,WPH}$ |
| Fuel preheater | $\dot{E}x_{30}+\dot{E}x_{21}=\dot{E}x_{24}+\dot{E}x_{31}+\dot{E}x_{D,FPH}$ |
| Air preheater | $\dot{E}x_{31}+\dot{E}x_{22}=\dot{E}x_{25}+\dot{E}x_{32}+\dot{E}x_{D,APH}$ |
| Fuel mixer | $\dot{E}x_{23}+\dot{E}x_{24}=\dot{E}x_{26}+\dot{E}x_{D,mixer}$ |
| Afterburner | $\dot{E}x_{27}+\dot{E}x_{28}=\dot{E}x_{29}+\dot{E}x_{D,afterburner}$ |
| **SOFC 3** |  |
| SOFC stack | $\dot{E}x_{42}^{ch}+\dot{E}x_{42}^{ph}+\dot{E}x_{41}^{ch}+\dot{E}x_{41}^{ph}=\dot{E}x_{43}^{ch}+\dot{E}x_{43}^{ph}+\dot{E}x_{44}^{ch}+\dot{E}x_{44}^{ph}+\dot{E}x_{D, SOFC}$ |
| Water pump | $\dot{E}x_{33}+W_{WP}=\dot{E}x_{36}+\dot{E}x_{D,WP}$ |
| Fuel compressor | $\dot{E}x_{34}+W_{comp}=\dot{E}x_{37}+\dot{E}x_{D,comp}$ |
| Air compressor | $\dot{E}x_{35}+W_{comp}=\dot{E}x_{38}+\dot{E}x_{D,comp}$ |
| Water preheater | $\dot{E}x_{45}+\dot{E}x_{36}=\dot{E}x_{39}+\dot{E}x_{46}+\dot{E}x_{D,WPH}$ |
| Fuel preheater | $\dot{E}x_{46}+\dot{E}x_{37}=\dot{E}x_{40}+\dot{E}x_{47}+\dot{E}x_{D,FPH}$ |
| Air preheater | $\dot{E}x_{47}+\dot{E}x_{38}=\dot{E}x_{41}+\dot{E}x_{48}+\dot{E}x_{D,APH}$ |
| Fuel mixer | $\dot{E}x_{39}+\dot{E}x_{40}=\dot{E}x_{42}+\dot{E}x_{D,mixer}$ |
| Afterburner | $\dot{E}x_{43}+\dot{E}x_{44}=\dot{E}x_{45}+\dot{E}x_{D,afterburner}$ |
| **SOFC 4** |  |
| SOFC stack | $\dot{E}x_{58}^{ch}+\dot{E}x_{58}^{ph}+\dot{E}x_{57}^{ch}+\dot{E}x_{57}^{ph}=\dot{E}x_{59}^{ch}+\dot{E}x_{59}^{ph}+\dot{E}x_{60}^{ch}+\dot{E}x_{60}^{ph}+\dot{E}x_{D, SOFC}$ |
| Water pump | $\dot{E}x_{49}+W_{WP}=\dot{E}x_{52}+\dot{E}x_{D,WP}$ |
| Fuel compressor | $\dot{E}x_{50}+W_{comp}=\dot{E}x_{53}+\dot{E}x_{D,comp}$ |
| Air compressor | $\dot{E}x_{51}+W_{comp}=\dot{E}x_{54}+\dot{E}x_{D,comp}$ |
| Water preheater | $\dot{E}x_{61}+\dot{E}x_{52}=\dot{E}x_{55}+\dot{E}x_{62}+\dot{E}x_{D,WPH}$ |
| Fuel preheater | $\dot{E}x_{62}+\dot{E}x_{53}=\dot{E}x_{54}+\dot{E}x_{63}+\dot{E}x_{D,FPH}$ |
| Air preheater | $\dot{E}x_{63}+\dot{E}x_{54}=\dot{E}x_{57}+\dot{E}x_{64}+\dot{E}x_{D,APH}$ |
| Fuel mixer | $\dot{E}x_{55}+\dot{E}x_{56}=\dot{E}x_{58}+\dot{E}x_{D,mixer}$ |
| Afterburner | $\dot{E}x_{59}+\dot{E}x_{60}=\dot{E}x_{61}+\dot{E}x_{D,afterburner}$ |
| **Exhausts of SOFC** |  |
| Mix 1 | $\dot{E}x_{16}+\dot{E}x_{65}=\dot{E}x_{66}+\dot{E}x_{D,mix 1}$ |
| Mix 2 | $\dot{E}x_{48}+\dot{E}x_{32}=\dot{E}x_{65}+\dot{E}x_{D,mix 2}$ |
| Mix 3 | $\dot{E}x_{66}+\dot{E}x_{64}=\dot{E}x_{67}+\dot{E}x_{D,mix 3}$ |
| **ORC** |  |
| Evaporator | $\dot{E}x_{67}+\dot{E}x_{69}=\dot{E}x_{68}+\dot{E}x_{70}+\dot{E}x_{D,evap}$ |
| Turbine | $\dot{E}x_{70}=\dot{E}x_{71}+W_{turbine}+\dot{E}x_{D,turbine}$ |
| Condenser | $\dot{E}x_{71}+\dot{E}x_{73}=\dot{E}x_{72}+\dot{E}x_{74}+\dot{E}x_{D,condenser}$ |
| Pump | $\dot{E}x_{69}+W_{pump}=\dot{E}x_{72}+\dot{E}x_{D,pump}$ |
| **PEM electrolyzer** |  |
| PEME | $\dot{E}x_{H_{2}O,input}+W_{PEME}=\dot{E}x_{H_{2},out}+\dot{E}x_{D, PEME}$ |
